# Prevalence of osteoporosis in patients awaiting unicompartmental knee arthroplasty: a cross-sectional study

**DOI:** 10.3389/fendo.2023.1224890

**Published:** 2023-09-11

**Authors:** Zhikun Zhuang, Changyu Huang, Xianyi Chen, Qiushi Wei, Jinhua Guo, Zhiqing Xu, Rongkai Wu, Zhaoke Wu, Fudong Xu

**Affiliations:** ^1^ Department of Orthopaedic Surgery, Quanzhou Orthopedic-traumatological Hospital, Quanzhou, China; ^2^ Quanzhou Medical College, Quanzhou, China; ^3^ Traumatology & Orthopedics Institute of Guangzhou University of Chinese Medicine, Guangzhou, Guangdong, China

**Keywords:** osteoporosis, unicompartmental knee arthroplasty, prevalence, treatment, bone mineral density

## Abstract

**Objectives:**

Osteoporosis may contribute to failure of unicompartmental knee arthroplasty (UKA), yet the prevalence of osteoporosis in the population awaiting UKA has not been adequately studied. The objectives of this study were to report the prevalence of osteoporosis in people awaiting UKA and the rate of anti-osteoporosis treatment, and to explore factors associated with osteoporosis prevalence in people awaiting UKA.

**Methods:**

Participants awaiting UKA from January 2019 to May 2023 were consecutively enrolled. Participants ‘ age, gender, BMI, knee K-L score, VAS score, history of previous DXA testing, history of anti-osteoporosis treatment, and possible underlying risk factors were recorded. All participants were given a dual-energy x-ray absorptiometry (DXA) test after the visit. The diagnosis of osteoporosis was made according to the World Health Organization criteria. Compare the prevalence of osteoporosis between people waiting for UKA and the general population. Risk factors associated with osteoporosis were analyzed using multiple linear regression and binary logistic regression models.

**Results:**

A total of 340 participants were included in the study, 259 in female and 81 in male, with a mean age of 63.53 years (range: 41-84 years), and all participants completed UKA and had DXA prior to UKA. The prevalence of osteoporosis was 40.88% (44.79% in female and 28.40% in male). The prevalence of osteoporosis was higher in female than in male (p<0.001). The prevalence of osteoporosis in the population waiting for UKA was significantly higher than that in the general population (p < 0.001). DXA testing was performed in 12.06% within 1 year prior to the visit. The percentage of those who had received anti-osteoporosis treatment was 20.59% (20.86% in osteoporosis, 22.39% in Osteopenia and 16.42% in normal bone mass). The correlation between age, gender, body mass index, visual analogue scale score and osteoporosis was statistically significant.

**Conclusion:**

Osteoporosis is common in people waiting for UKA, but screening and treatment rates are low. Female patients of advanced age and low weight combined with significant pain should be considered for osteoporosis screening and appropriate treatment before UKA.

## Introduction

Unicompartmental knee arthroplasty (UKA) has proven to be an effective treatment for anteromedial osteoarthritis (AMOA) of the knee that provides rapid recovery, fewer complications, and better function (1, 2). With the expansion of indications, patients meeting the indications for UKA could represent up to 50% of patients requiring total knee arthroplasty (TKA) (3, 4), which would be a huge number in an ageing society. The revision rate for UKA has been reported to be 2.1 times higher than that for TKA, and the reoperation rate is 1.4 times higher than that for TKA (1). In the available studies, the caseload of UKA by surgeons has been identified as an important factor contributing to the increased revision rate (5), but it seems easy for surgeons to overlook that osteoporosis may also contribute to UKA failure.

Osteoporosis is a systemic skeletal disease characterized by reduced bone mass and deterioration of bone tissue microarchitecture, which leads to increased bone fragility and an increased risk of fracture (6). Osteoporosis is common in patients awaiting total hip arthroplasty (THA) and TKA (7) and is associated with treatment failures that can lead to complications such as intraoperative fractures (8), periprosthetic fractures (9), aseptic loosening (10, 11), and prosthesis displacement (12). Treatment of osteoporosis before and after arthroplasty can reduce the revision rate of THA and TKA (13).

There are differences in the populations undergoing UKA and TKA, and it is generally accepted that patients undergoing UKA tend to be younger, healthier, and more energetic than those undergoing TKA. Nevertheless, the population of patients awaiting UKA is still at high risk for osteoporosis. Data from a large Danish study showed that the age of patients treated with UKA was approximately 65 years and rising (14). In the United States, women over 65 years of age are routinely referred for dual-energy X-ray absorptiometry (DXA) to screen for osteoporosis (15), suggesting that a large proportion of patients treated with UKA may have osteoporosis; however, the current emphasis by surgeons on osteoporosis in patients awaiting UKA seems to be insufficient. A reasonable speculation is that if a high percentage of osteoporosis is present in UKA patients, then attention to treatment targeting osteoporosis before and after UKA may reduce the failure rate of UKA. Therefore, it is important to know the prevalence of osteoporosis in patients awaiting UKA; however, there is a paucity of data on the prevalence of osteoporosis prior to UKA and few relevant studies.

Therefore, we conducted this study to explore: 1. What is the prevalence of osteoporosis in patients awaiting UKA? 2. How many patients with osteoporosis receive antiosteoporosis treatment prior to UKA? 3. What are the risk factors for osteoporosis in patients awaiting UKA.

## Materials and methods

### Study design and participants

This single-centre cross-sectional study was approved by our institutional ethics committee (EC of QMC [2020]009). All participants were recruited from our institution, a tertiary-care orthopaedic specialist hospital. The sample size was calculated based on the 33.3% (7) value of osteoporosis prevalence in the target population (we used the preoperative prevalence of total joint arthroplasty due to the unavailability of prior data), a confidence level of 95% and a margin error of 5%. The sample size required for this study was 340. The inclusion criterion was any patient over 18 years of age who awaited initial UKA. The exclusion criteria were traumatic knee osteoarthritis and patients who did not ultimately complete UKA. Our selection of the UKA procedure was based on the Oxford UKA criteria; specifically, UKA for patients with AMOA and spontaneous osteonecrosis of the knee (SONK) in whom patient age, weight, radiological factors, and medial cartilage ulceration of the lateral condyle were not considered contraindications (16). Patients seen at our hospital from January 2019 to May 2023 who were awaiting UKA were recruited consecutively, and all patients entering the cohort gave informed consent to the study.

### Data collection

The demographic parameters of the participants (age, gender, body mass index (BMI)) were recorded, and the Kellgren-Lawrence score was used for the knee X-ray assessment. The following information was also registered in the form of a questionnaire: visual analogue scale (VAS) score, possible risk factors for osteoporosis, including previous fracture, current smoking, secondary osteoporosis, alcohol 3 or more units/day, oral glucocorticoid intake for longer than three months in the past or at present, parent fractured hip, undergone DXA examination within 1 year before the visit, and antiosteoporosis treatment within 6 months before the visit.

All participants routinely underwent bone mineral density (BMD) measurements of the femoral neck, total hip, and L1-L4 spine within 1 month before the UKA procedure using DXA (Discovery-wi, Hologic, USA) according to the Chinese osteoporosis treatment guidelines. DXA was performed by two certified technicians. The T scores were determined with reference to the Chinese population reference database. Osteoporosis was diagnosed according to the World Health Organization (WHO) criteria (17): normal bone mass: T score > -1.0 SD, osteopenia: -2.5 SD < T score ≤ -1.0 SD, osteoporosis: T score ≤ -2.5 SD. A T score of ≤ -2.5 SD at any site was considered indicative of osteoporosis.

### Statistical analysis

The mean ± standard deviation ( ± S) was used to describe each parameter. The prevalence of osteoporosis in males and females, in males and females in different age groups (40-49, 50-59, 60-69, and >70 years), and in the general population and those waiting for UKA were analysed for statistical significance using chi-square tests. The data of the general population came from the newly reported epidemiological survey of osteoporosis in China (18). Smoothed curves for BMD of the lumbar spine, femoral neck and total hip by age and sex were plotted using GraphPad Prism 9.0.0 (GraphPad Software, USA). A multiple linear regression model was used to analyse the correlation of each variable with lumbar spine BMD in the total and female populations. Univariate analysis of each variable with osteoporosis in the total and female populations was performed using a binary logistic regression model. A multifactorial analysis of the binary logistic regression model was performed for gender, age, BMI, VAS, and glucocorticoid use. Bilateral p values of less than 0.05 were considered statistically significant. Statistical analyses were performed using SPSS 26.0 (IBM, Armonk, New York, USA).

## Results

### Demographics and indications in the population awaiting UKA

A total of 362 consecutive participants were recruited, excluding 2 cases of traumatic arthritis, 1 case of malignancy, and 19 cases converted to perform TKA, resulting in the inclusion of 340 cases. Among them, 307 participants were diagnosed with AMOA and 33 participants with SONK. There were 259 females and 81 males with a mean age of 63.53 years (range: 41-84 years) and a BMI of 24.35 ± 3.76 kg/m^2^. All participants completed UKA and underwent DXA prior to UKA. Demographic parameters are shown in **Table 1**.

**Table 1:** Demographic characteristics and prevalence of osteoporosis among participants.

	Total	Osteoporosis	Osteopenia	Normal
Age (years, $\bar{X}$ ± S, n, %)	63.53 ± 7.67, 340,100%	67.73 ± 6.48, 139,40.88%	62.10 ± 7.0, 134, 39.41%	57.64 ± 6.23, 67, 19.71%
40-49	45.88 ± 2.64, 8, 2.35%	46, 1, 12.50%	46.0 ± 3.0, 3, 37.50%	45.75 ± 3.20, 4, 50%
50-59	55.57 ± 2.56, 100, 29.41%	57.07 ± 2.06, 14, 14%	55.79 ± 2.39, 47, 47%	54.77 ± 2.67, 39, 39%
60-69	64.65 ± 2.84, 161, 47.35%	65.65 ± 2.56, 74, 45.96%	63.92 ± 2.91, 65, 40.37%	63.45 ± 2.48, 22, 13.66%
≥70	74.18 ± 3.52, 71, 20.88%	74.26 ± 3.91, 50, 70.42%	74.05 ± 2.55, 19, 26.76%	73.50 ± 0.71, 2, 2.82%
Gender (n, %)				
Male	81, 23.82%	23, 28.40%	29, 35.80%	29, 35.80%
Female	259, 76.18%	116, 44.79%	105, 40.54%	38, 14.67%
BMI (kg/m^2^, $\bar{X}$ ± S)	24.35 ± 3.76	22.95 ± 3.32	25.05 ± 3.57	25.84 ± 4.05
BMD (g/cm^2^, $\bar{X}$ ± S)				
L1-L4 spine	0.89 ± 0.19	0.75 ± 0.16	0.92 ± 0.10	1.13 ± 0.11
Femoral neck	0.68 ± 0.15	0.56 ± 0.08	0.70 ± 0.08	0.88 ± 0.09
Total hip	0.79 ± 0.23	0.68 ± 0.20	0.79 ± 0.24	0.98 ± 0.11
Received anti-osteoporosis treatment before operation (N, %)	70, 20.59%	29, 20.86%	30, 22.39%	11, 16.42%
VAS ( $\bar{X}$ ± S)	5.86 ± 1.11	6.24 ± 1.04	5.73 ± 1.10	5.30 ± 1.0
K-L score ( $\bar{X}$ ± S)	3.65 ± 0.48	3.60 ± 0.49	3.69 ± 0.46	3.64 ± 0.48
Previous fracture (n, %)	6, 1.76%	3, 2.16%	2, 1.49%	1, 1.49%
Ever-smoker (n, %)	37, 10.88%	16, 11.51%	10, 7.46%	11, 16.42%
Alcohol consumption (n, %)	24, 7.06%	12, 8.63%	7, 5.22%	5, 7.46%
Glucocorticoid use >3 months (n, %)	15, 4.41%	10, 7.19%	4, 2.99%	1, 1.49%
Parent Fractured Hip (n, %)	14, 4.12%	8, 5.76%	4, 2.99%	2, 2.99%
Diabetes (n, %)	40, 11.76%	18, 12.95%	14, 10.45%	8, 11.94%

BMI, body mass index; BMD, bone mineral density; VAS, visual analogue scale; K-L, Kellgren-Lawrence.

### Prevalence of osteoporosis in the population awaiting UKA

**Figure 1:**
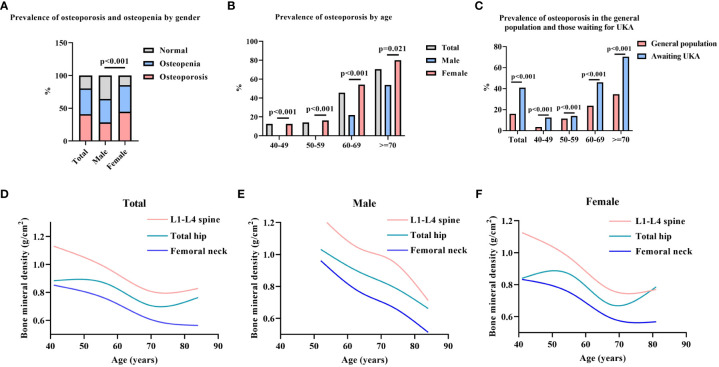
Prevalence of osteoporosis among participants. **(A)**. Prevalence of osteoporosis and osteopenia by gender; **(B)**. Prevalence of osteoporosis by age; **(C)**. Prevalence of osteoporosis in the general population and those waiting for UKA; **(D–F)**. Smoothed curves for BMD of the lumbar spine, femoral neck, and total hip by age in total, male, and female. Chi-square test was used for statistical analysis; The data of the general population came from the newly reported epidemiological survey of osteoporosis in China (18).

The percentages of osteoporosis, osteopenia and normal bone mass in the population waiting for UKA were 40.88%, 39.41%, and 19.71, respectively. The percentages in the male group were 28.40%, 35.80%, and 35.80%, respectively, and the percentages in the female group were 44.79%, 40.54%, and 14.67%, respectively (**Figure 1A**).

There was a significant difference in the prevalence of osteoporosis between males and females (p<0.001) (**Figure 1A**). The prevalence of osteoporosis in the total population was 12.5%, 14%, 45.96%, and 70.42% for ages 40-49, 50-59, 60-69, and >70 years, respectively (**Table 1**). The prevalence of osteoporosis was higher in females than in males at all ages (p<0.001, p<0.001, p<0.001, p=0.021, respectively) (**Figure 1B**). The prevalence of osteoporosis in the population waiting for UKA was higher than that in the general population in all age groups (p<0.001, p<0.001, p<0.001, p<0.001, respectively) (**Figure 1C**).

The BMDs of the participants’ L1-L4 spine, femoral neck, and total hip were 0.89 ± 0.19 g/cm^2^, 0.68 ± 0.15 g/cm^2^, and 0.79 ± 0.23 g/cm^2^, respectively (**Table 1**). In males, the decreasing trend of BMD with age was more stable, while female patients showed a sharp decrease in BMD between 60 and 70 years of age, which tended to moderate after 70 years of age (**Figures 1D–F**). In the total population, 20.59% received antiosteoporosis treatment before coming to our hospital, including 20.86% with osteoporosis, 22.39% with osteopenia, and 16.42% with normal bone mass (**Table 1**). DXA testing was performed in 12.06% (41/340) of patients within 1 year prior to the visit.

### Factors significantly associated with lumbar spine BMD

In multiple linear regression analysis, the following factors were significantly associated with lumbar spine BMD in the total population: gender (B: 0.191, 95% CI: 0.147 to 0.235), age (60-69 years, B: -0.188, 95% CI: -0.315 to -0.062; >70 years, B: -0.263, 95% CI: -0.393 to -0.134), BMI (underweight, B: -0.140, 95% CI: -0.258 to -0.022; overweight, B: 0.088, 95% CI: 0.048 to 0.128), VAS (B: -0.041, 95% CI: -0.055 to -0.027), and diabetes (B: 0.011, 95% CI: -0.038 to 0.060). In the female population, lumbar spine BMD was significantly associated with age (50-59 years, B: -0.105, 95% CI: -0.204 to -0.007; 60-69 years, B: -0.245, 95% CI: -0.342 to -0.147; >70 years, B: -0.325, 95% CI: -0.427 to -0.222), BMI (underweight, B: -0.199, 95% CI: -0.224 to -0.014; overweight, B: 0.079, 95% CI: 0.041 to 0.118), and VAS (B: -0.028, 95% CI: -0.042 to -0.013) (**Table 2**).

**Table 2:** Linear regression analysis of risk factors of L1-L4 spine BMD in population waiting for UKA.

	L1-L4 spine (Total, n=340)		L1-L4 spine (Female, n=259)	
	Regression coefficient (95%CI)	p-value	Regression coefficient (95%CI)	p-value
Gender	0.191 (0.147, 0.235)	<0.001	N/A	
Age (years)				
40-49	ref		ref	
50-59	-0.078 (-0.206, 0.050)	0.234	-0.105 (-0.204, -0.007)	0.037
60-69	-0.188 (-0.315, -0.062)	0.004	-0.245 (-0.342, -0.147)	<0.001
≥70	-0.263 (-0.393, -0.134)	<0.001	-0.325 (-0.427, -0.222)	<0.001
p for trend		<0.001		<0.001
BMI (kg/m^2^)				
<18.5 (underweight)	-0.140 (-0.258, -0.022)	0.020	-0.199 (-0.224, -0.014)	0.027
18.5–23.9 (normal weight)	ref		ref	
≥24.0 (overweight and obese)	0.088 (0.048, 0.128)	<0.001	0.079 (0.041, 0.118)	<0.001
p for trend		<0.001		<0.001
VAS	-0.041 (-0.055, -0.027)	<0.001	-0.028 (-0.042, -0.013)	<0.001
K-L score	-0.003 (-0.036, -0.029)	0.848	-0.008 (-0.041, 0.026)	0.654
Previous fracture	-0.003 (-0.124, -0.118)	0.965	0.009 (-0.111, 0.129)	0.884
Ever-smoker	0.013 (-0.046, 0.072)	0.662	0.082 (-0.034, 0.198)	0.164
Alcohol consumption	-0.054 (-0.120, 0.012)	0.108	-0.002 (-0.099, 0.094)	0.967
Glucocorticoid use >3 months	-0.049 (-0.131, 0.033)	0.244	-0.091 (-0.186, 0.004)	0.061
Parent Fractured Hip	-0.003 (-0.083, 0.077)	0.942	-0.015 (-0.099, 0.069)	0.731
Diabetes	0.011 (-0.038, 0.060)	0.018	0.010 (-0.038, 0.058)	0.684

BMI, body mass index; BMD, bone mineral density; VAS, visual analogue scale; K-L, Kellgren-Lawrence; CI, confidence interval; N/A, Not available.

### Factors significantly associated with osteoporosis

The total population was significantly associated with osteoporosis in a univariate analysis in a binary logistic regression model with gender, age, BMI, VAS, and history of glucocorticoid application. When the above factors were included in the multifactorial model for analysis, gender (OR: 4.397, 95% CI: 2.230 to 8.670), age (>70 years, OR: 16.667, 95% CI: 1.909 to 143.997), BMI (underweight, OR: 9.118, 95% CI: 1.128 to 73.733; overweight, OR: 0.445, 95% CI: 0.284 to 0.698), and VAS (OR: 1.712, 95% CI: 1.327 to 2.208) were significantly associated with osteoporosis (**Table 3**). In the female population, age, BMI, and VAS were significantly associated with osteoporosis in the univariate analysis of the binary logistic regression model, and when the above factors were included in the analysis of the multifactorial model, age (>70 years, OR: 28.0, 95% CI: 3.044 to 257.537), BMI (underweight, OR: 6.154, 95% CI: 0.745 to 50.824; overweight, OR: 0.360, 95% CI: 0.214 to 0.603), and VAS (OR: 1.565, 95% CI: 1.184 to 2.068) were significantly associated with osteoporosis in females (**Table 3**).

**Table 3:** Logistic regression analysis of risk factors of osteoporosis in population waiting for UKA.

	Total, n=340			Female, n=259		
	Univariable analysis (p-value)	Multivariable analysis (p-value)	OR (95% CI)	Univariable analysis (p-value)	Multivariable analysis (p-value)	OR (95% CI)
Female	0.010	<0.001	4.397 (2.230, 8.670)	N/A		
Age (years)	<0.001			<0.001		
40-49		ref			ref	
50-59		0.906	1.140 (0.130, 9.981)		0.781	1.361 (0.155, 11.946)
0-69		0.099	5.954 (0.716, 49.509)		0.051	8.273 (0.987, 69.325)
≥70		0.011	16.667 (1.909, 143.997)		0.003	28.0 (3.044, 257.537)
p for trend		<0.001	4.386 (2.878, 6.682)		<0.001	4.282 (2.708, 6.771)
BMI (kg/m2)	<0.001			<0.001		
<18.5 (underweight)		0.038	9.118 (1.128, 73.733)		0.092	6.154 (0.745, 50.824)
18.5–23.9 (normal weight)		ref			ref	
≥24.0 (overweight and obese)		<0.001	0.445 (0.284, 0.698)		<0.001	0.360 (0.214, 0.603)
p for trend		0.002	0.464 (0.287, 0.751)		0.001	0.385 (0.224, 0.661)
VAS	<0.001	<0.001	1.712 (1.327, 2.208)	<0.001	0.002	1.565 (1.184, 2.068)
K-L score	0.171			0.657		
Previous fracture	0.649			0.496		
Ever-smoker	0.757			0.828		
Alcohol consumption	0.349			0.983		
Glucocorticoid use >3 months	0.047	0.255	2.175 (0.571, 8.285)	0.118		
Parent Fractured Hip	0.214			0.331		
Diabetes	0.573			0.969		

BMI, body mass index; BMD, bone mineral density; VAS, visual analogue scale; K-L, Kellgren-Lawrence; CI, confidence interval; N/A, Not available; OR, odds ratio.

## Discussion

In this study, we observed that the prevalence of osteoporosis in the population awaiting UKA was significantly higher than that in the general population. Specifically, 80.29% of patients had bone mass abnormalities, including 40.88% with osteoporosis and 39.41% with osteopenia. This is similar to the results of a survey of bone mass abnormalities prior to TJA (86%) (7). Patients awaiting UKA were younger than those awaiting TKA (mean age: 63.5 years vs. 69.7 years) and had a relatively lower prevalence of osteoporosis (40.88% vs. 59.8%) compared to patients in a previous survey of the population awaiting TKA in China (19). However, a study from Europe that included older (mean age: 77.9 years) patients awaiting TKA found a significantly lower prevalence of osteoporosis (18.1%) as well as osteopenia (46%) than was revealed in our results (19), possibly due to geographical differences, dietary structure, genetics, and other factors; with some studies suggesting that postmenopausal bone loss is significantly higher in Asian females than in European females (20).

Only 12.06% of patients underwent DXA prior to their visit to our institution, suggesting inadequate screening for osteoporosis in patients awaiting UKA and that surgeons may not pay enough attention to the bone mass profile prior to UKA. This may be related to the lack of current data on the prevalence of osteoporosis prior to UKA. To the best of our knowledge, we were the first to report the prevalence of osteoporosis in Chinese patients awaiting UKA.

A total of 20.59% of patients received antiosteoporosis treatment prior to their visit to our hospital, which is similar to the preoperative TJA data (22%) (7). Although the rate of treatment for osteoporosis in patients awaiting UKA is severely underrepresented, it is still higher than that of osteoporosis patients in the general population, where only 1.4% of females and 0.3% of males with osteoporosis received antiosteoporosis treatment in a large study of a Chinese population (18). The relatively high treatment rate among those awaiting UKA may be related to the long course of AMOA, with most patients requiring UKA having repeated visits to community hospitals over the long course of the disease and possibly receiving antiosteoporosis treatment. However, surprisingly, 16.42% of patients with normal bone mass received antiosteoporosis treatment. We could not clarify whether this was a prophylactic treatment given based on consideration of osteoporosis risk factors; however, it exposes to some extent a possible overtreatment of osteoporosis in the normal population waiting for UKA. The lack of treatment rates for patients with osteoporosis and the possible overtreatment of patients with normal bone mass suggest a clear confusion about effective screening and accurate treatment of osteoporosis in the population awaiting UKA.

In this study, the BMD in the population of patients waiting for UKA tended to decrease with age (**Figures 1D, E**), and the prevalence of osteoporosis increased (**Figure 1B**), with females having a significantly higher prevalence than males. The significant association of age and gender with osteoporosis was likewise confirmed in logistic regression models, similar to that in the general population in China (18, 21, 22). In a multifactorial model analysis, we found a significant positive association between rising VAS scores and the prevalence of osteoporosis. Chronic joint pain is the most reported menopausal symptom among Asian women, and a survey from Singapore identified chronic joint pain as a risk factor for spinal osteoporosis (23), similar to our report. Chronic pain is associated with multiple factors, including comorbid underlying disease; we included the underlying disease in our multiple linear regression model and found no significant confounders. Therefore, DXA screening may be more necessary for patients with more severe pain awaiting UKA. BMI was also included in the multivariate analysis model, with a positive association with the prevalence of osteoporosis at BMI <18.5 kg/m^2^ and a negative association with osteoporosis at BMI >24.0 kg/m^2^. A lower BMI suggests an inadequate nutritional status, which may include insufficient intake of vitamin D as well as micronutrients, possibly contributing to osteoporosis; also, a low BMI suggests a relative lack of muscle strength, and a decrease in grip strength has been shown to be associated with osteoporosis (24). Therefore, greater emphasis should be placed on screening for osteoporosis in patients with a BMI <18.5 kg/m^2^.

There are some limitations to be noted regarding this study. First, this was a cross-sectional study, and the findings regarding risk factors for osteoporosis prevalence may be influenced by some uncertain confounding factors. Additionally, we were unable to verify the causal relationship between VAS and BMI and osteoporosis. Future prospective, controlled studies of this population are needed to better validate the above results. Second, this was a single-centre study, and despite our calculations of required sample size, China’s vast geography, large population, and diverse diet and lifestyle make our sample size still relatively inadequate. The implementation of a multiprovince, multicentre study may be able to address this limitation. Third, the proportion of non-Han populations in our region is low, so the suitability of the results of this study for non-Han populations in China needs to be further tested. As mentioned earlier, our findings may differ considerably from European populations, and this study may not be appropriate for non-Asian populations. Fourth, we did not obtain local bone density of the knee. This is because doing so could cause more radiation damage to the participants. Subchondral sclerosis due to knee osteoarthritis may enhance bone density, which does not reflect the overall bone density profile of the organism. Fifth, obtaining the history of previous fractures and the history of parental hip fractures with possible recall bias is unavoidable. Finally, whether there is a relationship between the presence of osteoporosis and failure of UKA is also unclear and requires a prospective study to further clarify.

## Conclusions

In conclusion, our study revealed that osteoporosis is common, under screened, and undertreated in people waiting for UKA. The development of osteoporosis may be related to age, gender, VAS score and BMI. Female patients of advanced age with low weight and notable pain should be considered for osteoporosis screening and appropriate treatment before UKA. Further studies are needed to explore the clinical benefits of perioperative antiosteoporosis treatment with UKA.

## Data availability statement

The original contributions presented in the study are included in the article/supplementary material, further inquiries can be directed to the corresponding author/s.

## Ethics statement

The studies involving humans were approved by Ethics committee of Quanzhou Medical College. The studies were conducted in accordance with the local legislation and institutional requirements. Written informed consent for participation was not required from the participants or the participants’ legal guardians/next of kin in accordance with the national legislation and institutional requirements.Written informed consent was obtained from the individual(s) for the publication of any potentially identifiable images or data included in this article.

## Author contributions

ZZ, CH and XC led the writing of the manuscript and contributed equally to this manuscript. Conceptualization, FX and ZW. Methodology, ZZ and Q-SW. Software, CH and JG. Validation, XC, and ZX. Formal Analysis, ZZ and RW. Investigation, CH and XC. Resources, XC and ZW. Data Curation, CH and ZX. Writing – Original Draft Preparation, ZZ, CH and XC. Writing – Review & Editing, FX and ZW. Visualization, ZZ and CH. Supervision, FX and ZW. Project Administration, ZZ. Funding Acquisition, FX and ZW. All authors contributed to the article and approved the submitted version.
